# Establishment and Evaluation of a Loop-Mediated Isothermal Amplification Assay for Rapid Detection of *Pseudomonas fluorescens* in Raw Milk

**DOI:** 10.3389/fmicb.2021.810511

**Published:** 2022-01-06

**Authors:** Yushan Bu, Wenjun Qiao, Zhengyuan Zhai, Tongjie Liu, Pimin Gong, Lanwei Zhang, Yanling Hao, Huaxi Yi

**Affiliations:** ^1^College of Food Science and Engineering, Ocean University of China, Qingdao, China; ^2^Key Laboratory of Functional Dairy, Co-constructed by Ministry of Education and Beijing Municipality, College of Food Science and Nutritional Engineering, China Agricultural University, Beijing, China; ^3^College of Food Science and Nutritional Engineering, China Agricultural University, Beijing, China

**Keywords:** *P. fluorescens*, raw milk, LAMP, detection, rapid

## Abstract

Raw milk is susceptible to microbial contamination during transportation and storage. *Pseudomonas fluorescens* producing heat-resistant enzymes have become the most common and harmful psychrophilic microorganisms in the cold chain logistics of raw milk. To rapidly detect *P. fluorescens* in raw milk, the protease gene *apr*X was selected as a detection target to construct a set of primers with strong specificity, and a loop-mediated isothermal amplification (LAMP) assay was established. The detection thresholds of the LAMP assay for pure cultured *P. fluorescens* and pasteurized milk were 2.57 × 10^2^ and 3 × 10^2^ CFU/mL, respectively. It had the advantages over conventional method of low detection threshold, strong specificity, rapid detection, and simple operation. This LAMP assay can be used for online monitoring and on-site detection of *P. fluorescens* in raw milk to guarantee the quality and safety of dairy products.

## Introduction

Dairy products are rich in all kinds of nutrients necessary for the human body and are easy to be digested and absorbed, so they are increasingly favored by consumers. Raw milk, the main material of dairy products, has a direct impact on the quality and safety of products. *Pseudomonas fluorescens* are types of psychrotrophic bacteria that can grow and reproduce at a low temperature. They exist widely in all links of dairy processing and production, which may cause potential hazards to raw milk (Vithanage et al., 2016). With the application of cold chain logistics in the dairy industry, *P. fluorescens* with a cold-tolerant characteristic occupy an advantage in the flora structure of raw milk and have become the main spoilage bacteria leading to the degradation of raw milk quality (Neubeck et al., 2015). In addition, some extracellular enzymes secreted by *P. fluorescens* can withstand high temperatures in the process of pasteurization and ultra-high temperature (UHT) sterilization (Alves et al., 2016). During the later storage of dairy products, the residual enzymes play a role in the hydrolysis of milk protein and fat, resulting in the reduction of nutritional value and adverse sensory changes, which shorten the shelf life (Stoeckel et al., 2016). The harm of *P. fluorescens* and their thermostable enzymes on dairy products has been widely studied (Barbano et al., 2006; Matéos et al., 2015; Zhang et al., 2020), which is considered to be one of the negative factors restricting the development of the dairy industry. Therefore, the monitoring and control of *P. fluorescens* in raw milk are significant to ensure the safety of dairy products.

The traditional counting method cannot detect *P. fluorescens* in raw milk quickly and specifically, which is difficult to meet the needs of real-time and on-site detection in the process of dairy production. In recent years, a large number of studies have been devoted to applying rapid detection technologies such as polymerase chain reaction (PCR), loop-mediated isothermal amplification (LAMP), and enzyme-linked immunosorbent assay (ELISA) (Hsieh et al., 2014; Hashimoto et al., 2016; Nagaraj et al., 2016; Madrid et al., 2021; Maier et al., 2021). PCR has been developed rapidly and is considered to be an accurate and reliable detection method. However, it is necessary to go through cumbersome temperature change procedures, and the results should be observed by electrophoresis in the process of PCR. As for the LAMP assay, at least four specific primers should be designed targeting six specific regions of the target gene (Niessen et al., 2013), and the amplification reaction will be completed under isothermal conditions for dozens of minutes with Bst DNA polymerase. The products of LAMP can be determined in several forms, such as agarose gel electrophoresis or turbidity measurement. In addition, a more simple and intuitive method is adding fluorescent dyes to the reaction system, and the final color difference of the system can be used to judge the occurrence of an amplification reaction directly (Wong et al., 2018). The LAMP method is superior in terms of its high specificity and low detection limit, and the equipment required for detection is simple. At present, the detection of *P. fluorescens* in raw milk based on LAMP is still in the exploratory stage. In this study, a set of specific LAMP primers was designed, and a rapid and efficient detection protocol of *P. fluorescens* in raw milk was established and evaluated, which was crucial to strengthening the quality control of dairy products.

## Materials and Methods

### Bacterial Strains and Culture Conditions

Milk-derived protease-producing *P. fluorescens* CICC 23250, CICC 23251, CICC 23253, and PF were the target strains in detection, which were inoculated into Luria–Bertani (LB) broth (QingDao Hope Bio-Technology, Shandong, China) containing 10 g of tryptone, 5 g of yeast extract powder, and 10 g of sodium chloride and were shaking cultured at 150 rpm at 25°C for 24 h. *Staphylococcus aureus*, *Escherichia coli*, *Listeria monocytogenes*, *Salmonella typhimurium*, *Pseudomonas aeruginosa*, and *Pseudomonas putida*, which are common contaminative bacteria in milk, were used to evaluate the specificity of the LAMP assay. *S. aureus* and *L. monocytogenes* were cultured in brain heart infusion (BHI) medium (Qingdao Hope Bio-Technology, Shandong, China) containing 10 g of peptone, 12.5 g of brain infusion powder, 5 g of heart infusion powder, 5 g of sodium chloride, 2 g of glucose, and 2.5 g of disodium phosphate at 37°C for 24 h, and the other four strains were cultivated with shaking in LB broth at 37°C for 24 h.

### Extraction of Bacterial DNA

DNA was extracted using the Bacterial Genomic DNA Extraction Kit (Solarbio, Beijing, China), and the extracted genome was used as a template in the LAMP amplification system.

### Design and Synthesis of a Primer Set for Loop-Mediated Isothermal Amplification

The complete sequence of the protease gene *apr*X of *P. fluorescens* was submitted to Clustal Omega^1^ for online multiple sequence alignment analysis. The part of the sequences with high conservatism were selected. Primer Explorer V5 software was used to design LAMP primers, including two outer primers (F3 and B3), two inner primers (FIP and BIP), and two loop primers (LF and LB). A primer set was synthesized by Shanghai Sangon Biotech. The primer sequences were listed in Table 1.

**TABLE 1:** Sequences of the primer set for *Pseudomonas fluorescens* protease gene *apr*X.

Primer	Sequence (5′–3′)
F3	GTACCTGACCAACAACAGCT
B3	GCTCTCGCTCCAGTAGCT
FIP	AGGGTATGGCCGATTTCGTGGGGAACAAGACCCCGGACCT
BIP	GCTCACCCTGGCGACTACAACGTGTAGCCGCGCGTGTC
LF	TGCCGGCCGTAGTTGTTC
LB	GGCAACCCGACCTACAACG

*F3 and B3 were the outer primers, FIP and BIP were the inner primers, and LF and LB were loop primers.*

### Optimization of Loop-Mediated Isothermal Amplification Reaction System

The initial added amount of each component and the reaction conditions in the LAMP system were referred to previous studies (Notomi et al., 2000; Nagamine et al., 2002) and modified slightly. The volume of the system was 25 μL, containing 12.5 μL of 2 × LAMP PCR Master Mix (Sangon Biotech, Shanghai, China), 0.5 μL of each outer primer, 2 μL of each inner primer, 1 μL of each loop primer, 2 μL of DNA template, 0.5 μL of Bst DNA polymerase, and double-distilled H_2_O (ddH_2_O) (Solarbio, Beijing, China). The system reacted at 65°C for 60 min. In order to achieve the optimum detection efficiency of the primer set, the LAMP detection system was optimized by taking *P. fluorescens* CICC 23250 as the representative strain. The optimization variables included the Bst DNA polymerase (Sangon Biotech, Shanghai, China) content (0.0, 0.1, 0.2, 0.4, 0.6, 0.8, 1.0, 1.2, 1.4, and 1.6 μL), Mg^2+^ (New England BioLabs, Beijing, China) concentration (0.0, 2.0, 4.0, 6.0, 8.0, 10.0, and 12.0 mmol/L), deoxynucleotide triphosphates (dNTPs) (Solarbio, Beijing, China) concentration (0.0, 0.4, 0.8, 1.2, 1.6, and 2.0 mmol/L), reaction temperature (60, 61, 62, 63, 64, and 65°C), and reaction time (20, 30, 40, 50, 60, and 70 min). An identical volume of ddH_2_O was used to replace the DNA template as a negative control. Bst DNA polymerase was inactivated at 80°C for 10 min to terminate the reaction. The LAMP reaction products were analyzed using 2% agarose gel electrophoresis at 100 V for 40 min, and amplification effects were judged by the gray values of electrophoretic bands with the help of Image J software.

### Specificity Evaluation of Loop-Mediated Isothermal Amplification Assay

Under the optimized reaction conditions, four strains of *P. fluorescens* and six strains of common pollutant bacteria were amplified with the designed primer set. The specificity of the LAMP assay system was analyzed by visualization and electrophoresis. One microliter of SYBR Green I (Solarbio, Beijing, China) was added into the LAMP reaction system, and the color change was observed directly to determine *P. fluorescens*. Another 5 μL of the reaction product was detected by 2% agarose gel electrophoresis at 100 V for 40 min to judge the specificity according to the generation of the characteristic map. *P. fluorescens* CICC 23250 was used as a positive control and ddH_2_O acted as a negative control.

### Detection Limit Measurement of the Loop-Mediated Isothermal Amplification Assay

Pure culture and artificially contaminated pasteurized milk with *P. fluorescens* CICC 23250 were taken for 10-fold gradient dilution. The DNA of *P. fluorescens* with different concentrations was extracted, and the concentration of bacteria was calculated by plate counting. Amplification was performed in the optimized LAMP system, and the detection limit of the LAMP system was determined by 2% agarose gel electrophoresis.

## Results

### Optimization of the Loop-Mediated Isothermal Amplification Reaction System

**FIGURE 1:**
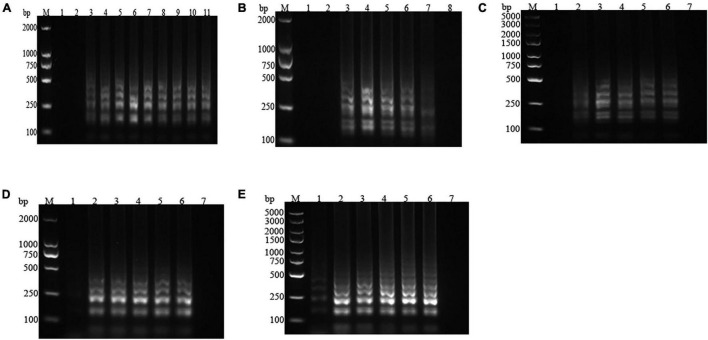
Effects of different Bst DNA polymerase contents, Mg^2+^ concentrations, deoxynucleotide triphosphates (dNTPs) concentrations, reaction temperatures, and reaction times on the electrophoretic bands of the loop-mediated isothermal amplification (LAMP) products. **(A)** Bst DNA polymerase content. *1*: negative control; *2*–*11*: 0.0, 0.1, 0.2, 0.4, 0.6, 0.8, 1.0, 1.2, 1.4, and 1.6 μL. **(B)** Mg^2+^ concentration. *1*–*7*: 0.0, 2.0, 4.0, 6.0, 8.0, 10.0, and 12.0 mmol/L; *8*: negative control. **(C)** dNTPs concentration. *1*–*6*: 0.0, 0.4, 0.8, 1.2, 1.6, and 2.0 mmol/L; *7*: negative control. **(D)** Reaction temperature. *1*–*6*: 60, 61, 62, 63, 64, and 65°C; *7*: negative control. **(E)** Reaction time. *1*–*6*: 20, 30, 40, 50, 60, and 70 min; *7*: negative control. *M* represents the D2000 plus DNA Ladder (Solarbio, Beijing, China).

The electrophoretic bands of products under different LAMP amplification conditions were shown in Figure 1. When Bst DNA polymerase was absent in the reaction system, there was no band present, while the band reached maximum brightness with Bst DNA polymerase content of 0.6 μL (Figure 1A). The results in Figure 1B revealed that, when the Mg^2+^ concentration was 4.0 mmol/L, an electrophoretic strip was detectable, which was brightest at a concentration of 6.0 mmol/L. dNTPs are the main materials for DNA synthesis determining the reaction degree of amplification. The brightest amplification band was observed at dNTPs concentration of 0.8 mmol/L (Figure 1C). It could be seen from Figure 1D that the brightness of the amplification band in lane 4 was more intense than that in other lanes, so the best reaction temperature was 63°C. For the reaction time, the band was visible at 20 min, but amplification was still incomplete. With the extension of the reaction time, there were no significant differences in the light intensity of the strips, indicating that the amplification was basically completed at 30 min. Therefore, 30 min was selected as the best reaction time to shorten the detection time (Figure 1E). In view of the above observations, the optimal LAMP assay system parameters were determined as 0.6 μL Bst DNA polymerase, 6.0 mmol/L Mg^2+^, 0.8 mmol/L dNTPs, and reaction temperature and time of 63°C and 30 min, respectively.

### Specificity of the Loop-Mediated Isothermal Amplification Assay

**FIGURE 2:**
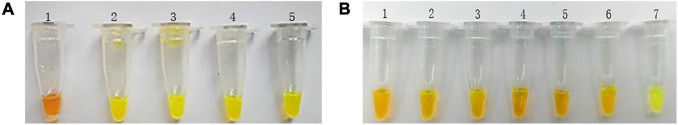
Visualization results of the loop-mediated isothermal amplification (LAMP) assay for *Pseudomonas fluorescens* and common contaminating bacteria in raw milk. **(A)** *P. fluorescens*. *1*: negative control; *2*–*5*: *P. fluorescens* CICC 23250, CICC 23251, CICC 23253, and PF. **(B)** Common contaminating bacteria. *1*–*6*: *Pseudomonas aeruginosa*, *Pseudomonas putida*, *Listeria monocytogenes*, *Staphylococcus aureus*, *Escherichia coli*, and *Salmonella typhimurium*; *7*: positive control.

**FIGURE 3:**
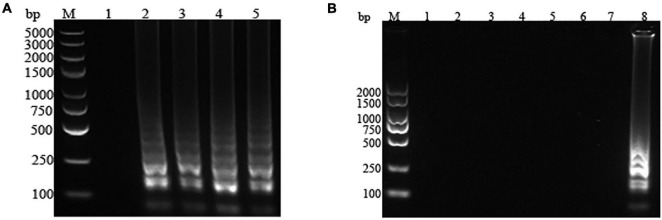
Electrophoresis map of the loop-mediated isothermal amplification (LAMP) assay for *Pseudomonas fluorescens* and common contaminating bacteria in raw milk. **(A)** *P. fluorescens*. *1*: negative control; *2*–*5*: *P. fluorescens* CICC 23250, CICC 23251, CICC 23253, and PF. **(B)** Common contaminating bacteria. *1*: negative control; *2*–*7*: *Pseudomonas aeruginosa*, *Pseudomonas putida*, *Listeria monocytogenes*, *Staphylococcus aureus*, *Escherichia coli*, and *Salmonella typhimurium*; *8*: positive control. *M* represents the D2000 DNA Ladder (Solarbio, Beijing, China).

The strains of *P. fluorescens* and common polluting bacteria (*S. aureus*, *E. coli*, *L. monocytogenes*, *S. typhimurium*, *P. aeruginosa*, and *P. putida*) in milk were amplified in the optimum LAMP reaction system. The results of the visualization and electrophoresis analysis were shown in Figures 2, 3, respectively. It was found that the amplification systems of the LAMP primers for the four *P. fluorescens* strains were positive (green), while presented negative (orange) for other contaminated strains, which suggested that the LAMP assay system would not be interfered by non-target strains (Figure 2). In Figure 3, the specific bands were observed for the four *P. fluorescens* strains, but no bands were found for the non-target strains. This indicated that the LAMP primer set had strong specificity for *P. fluorescens*, which was consistent with the results of visualization.

### Detection Limits of the Loop-Mediated Isothermal Amplification Assay

**FIGURE 4:**
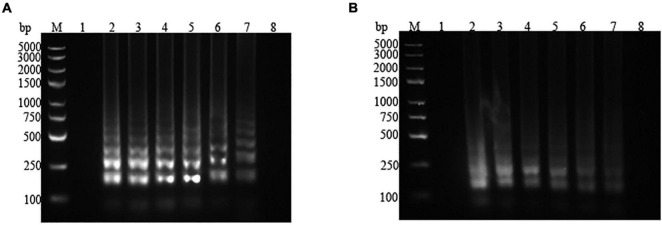
Electrophoresis map of the loop-mediated isothermal amplification (LAMP) assay for pure culture and artificially contaminated pasteurized milk with different concentrations of *Pseudomonas fluorescens.* **(A)** Pure culture. *1*: negative control (ddH_2_O); *2*–*8*: 2.57 × 10^7^, 2.57 × 10^6^, 2.57 × 10^5^, 2.57 × 10^4^, 2.57 × 10^3^, 2.57 × 10^2^, and 2.57 × 10^1^ CFU/mL. **(B)** Pasteurized milk. *1*: negative control (ddH_2_O); *2*–*8*: 3 × 10^7^, 3 × 10^6^, 3 × 10^5^, 3 × 10^4^, 3 × 10^3^, 3 × 10^2^, and 3 × 10^1^ CFU/mL. *M* represents the D2000 plus DNA Ladder.

LAMP amplification was performed in pure culture and contaminated pasteurized milk with different concentrations of *P. fluorescens*. The amplified products were subjected to 2% agarose gel electrophoresis. For *P. fluorescens* in pure culture, the concentrations ranging from 2.57 × 10^7^ to 2.57 × 10^2^ colony-forming unit (CFU)/mL could be amplified by the LAMP assay system. When the concentration of the reaction template was lower than 2.57 × 10^2^ CFU/mL, no band was detected, which suggested that the LAMP detection limit for pure cultured *P. fluorescens* was 2.57 × 10^2^ CFU/mL (Figure 4A). As shown in Figure 4B, the target amplification bands were observed in lanes 2–7, but no band was presented in lane 8, which demonstrated that the detection limit of the LAMP system for *P. fluorescens* in pasteurized milk was 3 × 10^2^ CFU/mL.

## Discussion

With the development and application of low-temperature preservation in the food industry, psychrophilic bacteria have become the main microorganisms leading to the corruption and deterioration of food. As the most common and hazardous cryophiles in raw milk, *P. fluorescens* can secrete extracellular enzymes with strong heat-resistant properties, which may reduce the nutritional value of food and cause foodborne diseases, affecting the quality and shelf life of dairy products seriously. At present, studies on the harmful effects of *P. fluorescens* and their thermostable enzymes to dairy products are mainly focused on pasteurized milk and UHT milk. Aging gels, protein hydrolysis, fat floating, and bitterness are common quality problems in UHT milk (Liu et al., 2007; Baglinière et al., 2013; Martin et al., 2018) and restrict the progress of the dairy industry. Therefore, it is essential to realize rapid detection of *P. fluorescens* in raw milk in order to ensure quality and safety in the dairy industry.

The traditional microbial counting method cannot meet the actual needs of the dairy enterprise due to its time-consuming process and inaccuracy. With the development of molecular biotechnology, an increasing number of rapid detection methods have been widely used for food microbe detection. LAMP assay relies on at least four especially designed primers and Bst DNA polymerase with strand-displacing activity to complete the amplification process at a constant temperature (Mori et al., 2013), which has a particular advantage over other methods. In the aspect of equipment, the reaction is carried out under thermostatic temperature, and a simple water bath can meet the requirement of temperature control without the need for expensive and sophisticated instruments. Moreover, a stabilized temperature reduces the time of temperature change, which greatly shortens the whole reaction process. With regard to the determination of the reaction products, it can be achieved using agarose gel electrophoresis and judged by the amount of flocculent turbidity or obvious color difference in the reaction system, which is more intuitive and reliable than the traditional plate counting method.

Owing to the short detection time, strong specificity, and simple operation (Notomi et al., 2015), the LAMP assay has the potential for on-site and real-time detection and has been widely used in microbial detection and identification (Wang et al., 2010; Duarte-Guevara et al., 2016). The LAMP assay or new detection approaches on the basis of LAMP have been reported to realize the rapid detection of microorganisms in food. Wang et al. (2013) established an *in situ* LAMP assay to detect *Vibrio parahaemolyticus* from seafood. It was found that the detection rates of the *in situ* LAMP, regular LAMP, and PCR were 100, 93.8, and 70.8%, respectively (Wang et al., 2013). In order to detect *L. monocytogenes* in food, primers targeting the genes *hly*A and *iap* were designed to construct the double LAMP (dLAMP) method. The results illustrated that the detection sensitivity was 10 fg DNA of *L. monocytogenes* per tube with reaction at 63°C for 15 min. At the same time, mineral oil and GoldView II dye were added into the system to avoid aerosol pollution and realize the visualization analysis (Wu et al., 2014). Kim et al. (2021) designed three LAMP primer sets for the detection of *Bifidobacterium longum* subspecies in probiotic products. The detection level was about 10^2^ CFU/mL, which could detect the target subspecies within 45 min specifically and sensitively (Kim et al., 2021). Restriction fragment length polymorphism (RFLP) combined with multiplex LAMP (mLAMP) was used to detect *Salmonella* spp. and *Shigella* spp. in milk. It was shown that the overall detection process could be completed in approximately 20 min. The sensitivity of mLAMP-RFLP for *Salmonella* and *Shigella* strains was up to 100 fg DNA per tube, and the detection limit in artificially contaminated milk was 5 CFU/10 mL (Shao et al., 2011).

The use of LAMP assay for detection in various food products has been reported (Sun et al., 2015; Spielmann et al., 2019; Liu et al., 2021). However, applying LAMP to detect *P. fluorescens* in raw milk is rare. The exploitation of a specific LAMP assay for detecting *P. fluorescens* in raw milk has attracted much attention. In our previous study, a LAMP assay system was established based on the conserved gene sequence of *P. fluorescens* 38 lipases, and we evaluated its application in raw milk (Xin et al., 2017). Although the reaction time of this LAMP system was 50 min, the dairy enterprise expected a faster detection speed. In this study, the primers were designed targeting the *P. fluorescens* protease gene, and the LAMP detection system was established and optimized. The results of the visual analysis and electrophoresis confirmed that the primer set had high specificity for *P. fluorescens*. For *P. fluorescens* in pure culture and pasteurized milk, the detection limits of the LAMP assay were 2.57 × 10^2^ and 3 × 10^2^ CFU/mL, respectively, which were applicable for the detection of *P. fluorescens* in raw milk during the actual production process. It was worth mentioning that, compared with the previous study (Xin et al., 2017), the addition of loop primers shortened the detection time to 30 min and improved the efficiency significantly.

## Conclusion

A LAMP assay system for rapid detection of *P. fluorescens* in raw milk was established and evaluated. The optimal reaction conditions were 0.6 μL Bst DNA polymerase, 6.0 mmol/L Mg^2+^, 0.8 mmol/L dNTPs, and 63°C reaction temperature. The LAMP detection system had strong specificity and low detection limit. Compared with the existing detection methods, the detection process of this LAMP assay could be completed in 30 min, which was significant in strengthening the quality control of dairy products.

## Data Availability Statement

The original contributions presented in the study are included in the article/supplementary material, further inquiries can be directed to the corresponding author/s.

## Author Contributions

YB and WQ conducted the experiments. YB wrote the manuscript. ZZ, TL, and PG supervised the experiments. LZ, YH, and HY revised the manuscript. All authors contributed to the article and approved the submitted version.

## Conflict of Interest

The authors declare that the research was conducted in the absence of any commercial or financial relationships that could be construed as a potential conflict of interest.

## Publisher’s Note

All claims expressed in this article are solely those of the authors and do not necessarily represent those of their affiliated organizations, or those of the publisher, the editors and the reviewers. Any product that may be evaluated in this article, or claim that may be made by its manufacturer, is not guaranteed or endorsed by the publisher.

## Footnotes

1
www.ebi.ac.uk/clustalw
